# Health Literacy and Health Conditions at the Intersections of Gender and Race in Later Life

**DOI:** 10.1093/geroni/igab046.3153

**Published:** 2021-12-17

**Authors:** Takashi Yamashita, Darren Liu, Betty Burston, Jennifer Keene

**Affiliations:** 1 University of Maryland, Baltimore County, Baltimore, Maryland, United States; 2 Des Moines University, Des Moines, Iowa, United States; 3 University of Nevada, Las Vegas, Las Vegas, Nevada, United States

## Abstract

The benefits of health literacy are well-documented. Health literacy is a set of skills to locate, understand, and use health-related information to make optimal health decisions. However, relatively less is known about the long-term relationship between health literacy and overall health conditions among older adults. Additionally, health literacy and health at the intersection of gender and race/ethnicity, rather than gender and race separately, are yet to be investigated. This study analyzed sub-samples (n = 1,260 adults age 50+) of the 2010 Health and Retirement Study (HRS) health literacy module data, and the 2012, 2014, and 2016 HRS data to examine the trajectories of health based on eight physical and mental conditions (0-8 points: better-worse) among older adults. Latent growth curve mixture models were used to investigate the changes in health and six groups defined by gender (women and men) and race/ethnicity (White, Black, and Hispanic). Results showed that overall health deteriorated over time (latent-slope = 0.19, p < 0.001) but the trajectories were diverse (latent-slope variance = 0.06, p < 0.001). Greater health literacy (0-5 points: worse-best scaling), which was measured with a validated scale, was associated with better overall health only among White women and men. Notably, White women received the baseline health benefits (b = -0.20, p < 0.05) from health literacy whereas Black women (b = 0.09, p > 0.05) did not [Δb = 0.09 -(-0.20) = 0.29, p < 0.05]. Other detailed comparisons, theoretical explanations, and public health policy implications for diverse older populations were evaluated.

